# Changing clinical significance of oocyte maturity grades with advancing female age advances precision medicine in IVF

**DOI:** 10.1016/j.isci.2023.107308

**Published:** 2023-07-11

**Authors:** Cari Nicholas, Sarah Darmon, Pasquale Patrizio, David F. Albertini, David H. Barad, Norbert Gleicher

**Affiliations:** 1 Center for Human Reproduction, New York, NY, USA; 2 Department of Obstetrics, Gynecology, and Reproductive Sciences, University of Miami, Miller School of Medicine, Miami, FL, USA; 3 Bedford Research Foundation, Bedford, MA, USA; 4 Foundation for Reproductive Medicine, New York, NY, USA; 5 Stem Cell Biology and Molecular Embryology Laboratory, The Rockefeller University, New York, NY, USA; 6 Department of Obstetrics and Gynecology, Medical University of Vienna, Vienna, Austria

**Keywords:** Women’s health, Reproductive medicine, Cell biology

## Abstract

In current IVF practice, metaphase-2 (M2) oocytes are considered most efficient in producing good quality embryos. Maximizing their number at all ages is standard clinical practice, while immature germinal vesicle (GV) oocytes are mostly automatically discarded. We present preliminary evidence that oocyte maturity grades with advancing age significantly change in their abilities to produce good quality embryos, with M2 oocytes significantly declining, GV oocytes improving, and M1 oocytes staying the same. These data contradict the over-40-year-old dogma that oocyte grades functionally do not change with advancing age, supporting potential changes to current IVF practice: (1) Stimulation protocols and timing of oocyte retrieval can be adjusted to a patient’s age and ovarian function. (2) In older and younger women with prematurely aging ovaries, GV oocytes may no longer be automatically discarded. (3) In some infertile women, rescue *in vitro* maturation of immature oocytes may delay the need for third-party egg donation.

## Highlights

• Oocyte maturity grades change with age in ability to produce good quality embryos
• M2s decline, GV oocytes improve, and MIs remain unchanged
• Rescue *in vitro* maturation may, therefore, increase embryo numbers
• These findings, therefore, offer a step toward better precision medicine in IVF

## Graphical abstract

**Figure:**
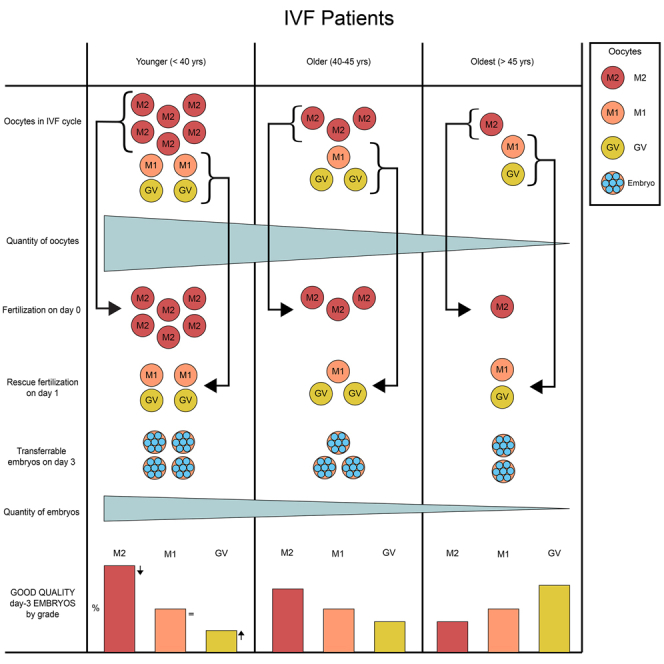

## Introduction

**Figure 1:**
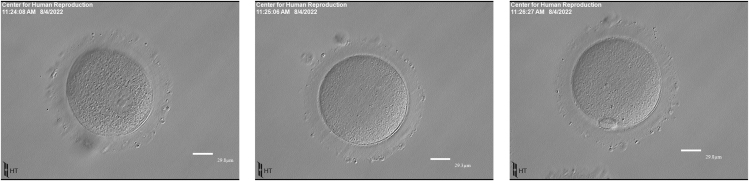
Oocyte maturity grading From left to right, GV oocyte, characterized by an intracytoplasmic nucleus (germinal vessel); M1 oocyte presents with neither a germinal vesicle nor polar body; M2 oocyte presents with a typical polar body. The scale bar is measured in μm.

Among only very few undisputed issues regarding *in vitro* fertilization (IVF), the maturity grading of retrieved oocytes into metaphase 2 (M2, mature), metaphase 1 (M1, mildly immature), and germinal vesicle stage (GV, very immature) (Figure 1) is, likely, least contested. IVF cycles characteristically produce oocytes at different maturity grades. Since M2 oocytes are widely perceived as the most efficient in producing good quality embryos, the best pregnancy rates, and the best live birth rates, practically all IVF programs strive to maximize the percentage of M2 oocytes and to minimize especially very immature GV oocytes. Though some embryology laboratories in recent years have attempted overnight rescue-*in vitro* maturation for M1 and GV oocytes,^1^ most, because of poor success, automatically discard especially GV oocytes.

Therefore, IVF programs for all patient ages routinely attempt to maximize M2 and minimize GV oocytes. Such prioritization of M2 oocytes was also supported by a recent study in young women under age 36, which demonstrated that M1 oocytes that matured during *in vitro* maturation more quickly into M2 oocytes offered better IVF cycle outcomes than slower maturing M1s^2^ and by the fact that, after fertilization, embryo quality is almost completely a reflection of oocyte competences.^3^ Therefore, maximizing M2 oocyte yields at all ages and minimizing GV numbers for many years was also standard practice at our fertility center.

Our center serves a very adversely selected patient population, characterized by older age (median persistently >43 years, with national US IVF data for the same years stable around age 36), by unusually high numbers of prior IVF failures, and in over half of the cases, by patients previously advised that third-party egg donation represented their only chance of conception. Over time, we learned that this patient population demanded deviations from many standard IVF treatments that they, often, previously already failed.

Like most IVF centers, we have been observing a slow but steady decline in pregnancy and live birth rates in patients with advancing age. However, by also treating large numbers of older women than most other IVF clinics with the use of autologous oocytes, we also had the opportunity to notice that this decline accelerated after age 43. In trying to understand why that was the case, we discovered that follicles luteinized with advancing age earlier and earlier and, therefore, at progressively smaller lead follicle sizes. To avoid premature luteinization, oocyte retrievals, therefore, had to be scheduled at progressively smaller lead follicle sizes, a process we gave the acronym HIER (Highly Individualized Egg Retrieval).^4^^,^^5^

HIER was implemented at our center in 2014 and allowed us to diminish the sudden decline in pregnancy and live birth rates after age 43, best documented by a clear extension in the age of women still conceiving and delivering with the use of autologous oocytes. Therefore, we have been able to extend the age of our center’s (and likely the world’s) oldest woman to so far have delivered a child with the use of autologous eggs to two weeks short of her 48 th birthday at the time of embryo transfer. Consequently, her lead follicle at the time of trigger was only at 12 mm average diameter,^6^ while at almost all IVF centers worldwide, ovulation triggers to this day, independently of age, are still routinely given at 18-22 mm. HIER, moreover, does not significantly increase the proportions of M2, M1, and GV oocytes obtained in such IVF cycles.^4^^,^^5^

Practicing HIER experimentally since 2014 and routinely since 2016, we also increasingly recognized the importance of egg quality in predicting embryo quality and, therefore, IVF cycle outcomes.^7^^,^^8^^,^^9^ Concentrating on egg quality in analyzing failed IVF cycles, raised our suspicion that oocyte maturity grades may have different capacities to produce good quality embryos at different ages and, ultimately, led to this here-presented study, in which we restrospectively age-stratified, analyzed M2, M1, and GV oocytes in their ability to form good quality, transferrable, cleavage-stage embryos.

## Results

Retroactively investigating 150 consecutive IVF cycles performed at our center during 2021–2022, only donor-egg cycles and cycles in which no oocytes were retrieved were excluded. A total of 863 oocytes were retrieved in 103 women in 150 IVF cycles at median age 43 (range 32–52 years). Reflecting their poor prognosis beyond their advanced ages, median FSH and AMH were 9.0 mIU/mL and 0.643 ng/mL, respectively. Women under age 40 produced 265 oocytes, 161 M2s, 47 M1s, and 57 GV oocytes. Between ages 40–45 years, women produced 544 oocytes, 375 M2s, 89 M1s, and 80 GVs. Finally, above age 45, 54 oocytes were obtained, 35 M2s, 9 M1s, and 10 GVs. Interestingly, the percentages of M2, M1, and GV oocytes did not differ statistically between age groups, confirming earlier reports that earlier egg retrievals with HIER in older women do not to significant degrees increase the retrieval of immature oocytes.^4^^,^^5^

### IVF cycle stimulation protocol

Whatever the underlying cause, if androgens are low and/or if their sex hormone binding globulin (SHBG) is abnormally high, IVF cycles are initiated only after androgens and SHBG have normalized after pre-supplementation with dehydroepiandrosterone (DHEA, 25 mg TID, various manufacturers) for at least 6–8 weeks prior to IVF cycle start.^10^

Ovarian stimulation in almost all cases involved 300–450 I.U. of an FSH product and 150 I.U. of an hMG product (manufacturer varies per patient insurance). With especially low ovarian reserve, patients also receive clomiphene citrate (100 mg) for 5 days, starting on day 2 of menses.

### Highly Individualized Egg Retrieval (HIER)

In contrast to most IVF centers which trigger IVF cycles at lead follicle sizes between 18 and 22 mm, our center since 2014 practices HIER.^4^^,^^5^ This means that, primarily based on age and other cycle parameters, patients are ovulation-triggered at much smaller lead follicle sizes. How lead follicle sizes at ovulation trigger changed with advancing age in this here-presented study, is demonstrated in Table 1, revealing a significant decline of average lead follicle sizes at ovulation trigger from 18.4 ± 3.4 mm below age 40 to 14.4 ± 3.3 mm above age 46 (p < 0.001).

**Table 1:** Lead follicle size at ovulation trigger at different ages in investigated patients

<40 years		40-45 years		>45 years		p-value
n	mm	n	mm	n	mm	
34	18.4 ± 3.4	90	16.8 ± 2.7	26	14.4 ± 3.3	<0.001

n, number; The table demonstrates the progressively smaller lead follicle sizes with advancing female age at which patients were triggered.

Since triggers are given early, the risk of premature spontaneous ovulation almost no longer exists. Consequently, HIER cycles do not require either GnRH agonists or antagonists during hyperstimulation with gonadotropins to prevent premature ovulation. Here-investigated women, therefore, received neither.

Since percentages are by definition not normally distributed, both parametric and non-parametric statistical analyses were performed yielding almost identical results, with results obtained with the non-parametric Kruskal-Wallis test reported here: With advancing age, M2 oocytes produced significantly declining percentages of good quality transferrable embryos, from 63.8 ± 34.9% under age 40 to only 27.4 ± 40.2% at ages over 46 (p = 0.003), while GV oocytes over this time span improved from 2.0 ± 8.1% to 50.0 ± 44.7% (p = 0.012). Interestingly, M1 oocytes remained statistically unaffected (p = 0.754) (Table 2).

**Table 2:** Percentage of IVF cycles producing transferrable day 3 embryos with advancing age

Women (%) with transferrable embryos by age groups							
	<40 years		40-45 years		>45 years		p-value
	n	%	n	%	n	%	
M2	31	63.8 ± 34.9	83	53.1 ± 34.5	21	27.4 ± 40.2	0.003
M1	20	49.4 ± 46.9	45	45.7 ± 43.0	7	57.1 ± 53.5	0.754
GV	17	2.0 ± 8.1	45	22.2 ± 40.4	6	50.0 ± 44.7	0.012

n, number; The table presents data on 135 cycles that had M2 oocytes retrieved. An additional 15 cycles that entered the study had no M2 oocytes retrieved for a total of 150 cycles. 72/150 cycles had M1 and 68/150 had GV oocytes retrieved. As the table demonstrates, percentages of IVF cycles with transferrable embryos changed significantly with advancing female age in M2 and GV oocytes, declining in M2 oocytes (p = 0.003) and improving in GV oocytes (p = 0.012), while the effectiveness of M1 oocytes remained the same (p = 0.754, all by Kruskal-Wallis Test).

Since M1 and GV oocytes at our center routinely undergo rescue *in-vitro* maturation,^1^ Table 3 summarizes the procedure’s effectiveness with advancing age based on whether fertilization was normal (defined by the presence of 2 pronuclei 16–18 h after ICSI) or abnormal (presence of no, one, or more than 2 pronuclei): Though GV oocytes especially demonstrated a positive trend with advancing age in percentages of increasingly successful fertilization (p = 0.097), likely due to small numbers, neither group of oocytes reached statistical significance. The table also demonstrates that abnormal fertilization did not increase with successful fertilization. GV oocytes were also the only maturity grade demonstrating an improving trend in percentages of cleaving embryos with advancing age, though likely, again because of small case numbers, not reaching significance (50.0 ± 70.7% below age 40; 92.3 ± 27.7% at 40–45 years, and 100.0 ± 0.0% above age 46).

**Table 3:** Normal and abnormal fertilization rates in women at varying ages

	<40 years		40–45 years		>45 years		p-value
	n	%	n	%	n	%	
**Normal fertilization rates (%)**							

MII	31	74.9 ± 24.6	83	69.8 ± 34.0	22	62.5 ± 44.8	0.431
MI	15	74.9 ± 36.7	34	75.2 ± 38.7	5	100.0 ± 0.0	0.359
GV	3	33.3 ± 28.9	17	70.6 ± 43.5	5	80.0 ± 44.7	0.314

**Abnormal fertilization rates (%)**							

MII	31	6.6 ± 17.5	83	6.7 ± 16.5	22	9.1 ± 25.1	0.856
MI	15	4.9 ± 13.0	34	3.7 ± 17.6	5	0.0 ± 0.0	0.834
GV	3	16.7 ± 28.9	17	25.0 ± 39.5	5	0.0 ± 0.0	0.383

n, number; The table demonstrates no statistically significant changes with advancing female age in normal or abnormal fertilization rates, though GV oocytes demonstrate a trend toward better normal and, possibly, lower abnormal fertilization. Further larger studies will be required to clarify this point.

## Discussion

Following up on a hypothesis developed from observations of failed IVF cycles in a weekly clinical conference, this study produced surprising data in demonstrating with advancing female age, a significant shift in the efficiency of M2, M1, and GV oocytes in producing transferrable good-quality cleavage-stage embryos: M2 oocytes lost in efficiency, while GV oocytes to a remarkable degree improved their effectiveness of producing good quality embryos.

These findings are especially remarkable because they were obtained in patients universally treated with HIER and, therefore, triggered and retrieved much earlier than IVF patients would be triggered and retrieved in most IVF centers in the world. We previously reported that such earlier retrievals do not significantly increase percentages of immature oocytes,^4^^,^^5^ a finding in this study again confirmed and, in itself, suggesting that oocyte maturation significantly changes with advancing female age.

That very early oocyte retrievals did not significantly change the distribution of M2, M1, and GV oocytes in cycle cohorts, is an important physiologic observation that was discovered only relatively recently.^4^^,^^5^ Thus, this finding was expected. However, we were surprised by the observation that despite at times very early retrievals at lead follicle sizes as small as 11-12 mm, many M2 oocytes were still over-matured and, therefore, did progressively worse in producing good quality transferrable embryos with advancing female age.

M1 oocytes remained more or less constant in their performance but, as a second surprise, very immature GV oocytes with advancing female age significantly improved in their production of good-quality embryos. By demonstrating at both extremes of maturity contrasting trends between M2 and GV oocytes, this study also confirmed our earlier observation^4^^,^^5^ that the metabolism in follicles appears to speed up with advancing age. This conclusion was further supported by the observation made for the first time here that some GV oocytes during overnight incubation in standard culture media matured within a very short time period from a GV to an M2s grading that allowed for fertilization with one day delay. Due to this delay in fertilization, by day 3, the day of embryo transfer, these embryos would, therefore, be expected to demonstrate only a 2–4-cell day 2 phenotype. As yet another surprise, and as further evidence for the speeding up of maturing processes at older female ages, most so-fertilized embryos by day 3, however, demonstrated expected 6–8-cell phenotypes.

What causes such quick maturation is as of this point unknown, but at least one study in young oocyte donors suggested that cycle length is an indicator of oocyte quality and ovarian reserve with shortening cycles reflecting declining fecundity.^7^ Moreover, lead follicle sizes prior to spontaneous ovulation also appear to decline with advancing age, reported to be 19.6mm–21.3 mm between ages 21 and 36, but only 16.7 mm between ages 37 and 45.^8^ Lead follicle sizes at spontaneous ovulation above age 45 have not been reported, but would be expected to continue to decline.

By triggering ovulation at smaller and smaller lead follicle sizes progressively earlier, HIER, therefore, appears to mimic nature. Nature is also universally characterized by the loosening of inhibitory physiological mechanisms with advancing age. The usually controlled and slow recruitment of dormant primordial follicles into maturation represents a good example, well demonstrated in human ovarian transplants when interruption of restraining pathways caused by surgery leads to sudden explosive and massive recruitment of follicles.^9^

It is important to emphasize that in this study, reported outcomes as of this moment cannot be generalized because they were obtained in very adversely selected infertility patients at ages where most IVF centers no longer treat infertile women with autologous oocytes. Moreover, investigated patient groupings were not large enough to allow for separate determinations by race and/or ethnicity. Therefore, the aforementioned observations may have different impacts on different races and/or ethnicities. Since women in this age group usually are only offered conception through third-party egg donations, here-presented findings may nevertheless have translational implications: As our study group under age 40 well demonstrated, *in vitro* rescue maturation of especially GV oocytes, in such young ages are only rarely successful. Understandably, most IVF laboratories, therefore, automatically dispose of such oocytes. However, our study here demonstrated that, especially in oldest patients over age 45, GV oocytes produce good-quality cleavage-stage embryos in surprising numbers. Since the numbers of transferrable embryos, after female age, are the second-most important predictor of clinical IVF outcomes,^10^ it appears reasonable to assume that improved utilization of *in vitro*-matured GV oocytes may add a few percentage points to currently still very poor pregnancy and live birth rates in this patient age group.

This, of course, will have to be confirmed in future studies of larger patient populations. This study, moreover, had to rely as a primary endpoint on percentages of good quality embryos. To confirm the here-presented conclusions further, not only larger follow-up studies will be required, but outcomes will have to be followed through to the establishment of pregnancy and live births. Even confirmation of only a few additional percentage points in live birth rates will save substantial numbers of older fertility patients from having to give up on the use of their own oocytes in favor of third-party egg donations. We hope that this study will not only encourage further research into the underlying physiological mechanisms for the aforementioned observations, but will also encourage more IVF clinics to offer older women IVF cycles with the use of autologous oocytes, so that pregnancy and live birth data in older women, finally, can become available in sufficient numbers.

The here-presented study raises important additional questions: What defines the oocyte quality parameter (or parameters) that appear to decline in M2 oocytes with advancing age, yet in GV oocytes overnight appear to improve and result in seemingly producing good quality embryos? Also, how can these qualities potentially be even further enhanced? Does this phenomenon exist in selected oocytes of all older women or only in some? Are these processes the same in older women and younger women with premature ovarian aging (POA)?^11^ Is there an age threshold for the induction of this process or is it a gradual process, as observed in progressively early premature luteinization of follicles with advancing age?^4^^,^^5^ If the latter were to be the case, the aforementioned observations would suggest that not only the follicular metabolism speeds up with advancing female age, but also the nuclear and/or cytoplasmic maturation of oocytes.

That this, indeed, may be the case is supported by molecular and transcriptomic analysis of cumulus cells from oocytes of women of advanced reproductive age which suggested that follicular vascular hypoxia stresses the complete follicular environment.^12^ Here-presented data not only proposes that the clinical potential of oocytes at different maturity grades changes with advancing female age, but that what happens is an acceleration of metabolic processes not only in the microenvironment of the follicle but, likely, also in oocytes and/or in the communications between oocytes and their follicular microenvironment of cumulus and granulosa cells.^13^

As noted, our center in a weekly conference routinely reviews failed IVF cycles. Special attention is given to ovarian stimulation, ovulation-trigger timing defined by HIER,^4^^,^^5^ oocyte quality,^14^^,^^15^^,^^16^ and embryo quality by standard criteria that have been in place for decades.^17^ It was during these retroactive cycle reviews that we for the first time suspected that ovarian aging may affect the performance of oocyte maturity grades in older women and, possibly, also in younger women with POA, by some also called occult primary ovarian insufficiency (oPOI).^18^ These clinical observations then provided the impetus for the here-presented study.

If further confirmed, and if at least some of the above-mentioned additional questions can be answered, the data reported here have the potential of pointing IVF toward a new era of precision medicine, in which IVF cycles are no longer driven by uniform protocols but carefully individualized. This study also raises questions about the currently still almost universal worldwide practice of automatically discarding GV oocytes without attempts at rescue maturation. Finally, the results of this study, potentially, also encourage change in how third-party egg donations are utilized in contemporary IVF practice, as after ages 42–43, most IVF centers currently refuse the utilization of autologous oocytes,^6^^,^^19^ though a very recent study again refuted^20^ the widely quoted rationale for refusal of treatment with autologous oocytes beyond ages 42–43 is that pregnancy and live birth chances are too low. A secondary argument is that young donor eggs, of course, offer much better pregnancy and live birth rates.

The aforementioned observations do not apply to young infertility patients with normal functional ovarian reserve. A recent study in such young and unselected women undergoing IVF, indeed, suggested that follicle sizes under 12.5 mm at the time of retrieval (likely representing trigger sizes of 9-11 mm) rarely result in good-quality blastocysts.^21^

Lead follicle sizes at trigger are at our center in principle determined by patient age: Like in most IVF centers, up to age 40, in women with normal functional ovarian reserve, the recommended size is 18-22 mm. If women at such young ages, however, suffer from POA, they also become eligible for HIER. By age 43, the lead follicle size at trigger drops to 16 mm, and to 12–14 mm by age 45. Following prior cycle reviews, those criteria are, however, in previously noted weekly conferences often adjusted, considering such additional factors as speed of follicle growth, estradiol rise, as well as progesterone levels, and, of course, review of the quality of obtained oocytes and embryo development. Over two-thirds of adjustments trend toward earlier retrievals, while changes to later retrievals are rare.

Since M2 oocytes in most embryo cohorts are in the majority, they must remain the primary treatment target; but an important goal must also be to prevent them from over-maturing. This may require even earlier ovulation triggers and oocyte retrievals than current HIER practice demands. Here-presented study suggests that this approach may be surprisingly successful with M1 and, especially, with GV oocytes; but with M2 oocytes HIER proved to be less successful. M2 oocytes, however, even in oldest patients must continue playing an important role because, even with HIER, they still are a majority of oocytes in most embryo cohorts produced in an IVF cycle. Surprisingly good outcomes with M1 and GV, moreover, suggest that when in doubt, earlier retrievals may almost always be the best approach.

Our findings regarding GV oocytes were especially unexpected since, as of this point, they were the result of only simple overnight culture in standard culture media, and without additional supplementations. Rescue *in vitro* maturation likely, can be further improved with more specialized culture media, - a possibility already under investigation.

The ultimate question is, however, as of this point how small one can go in lead-follicle sizes? Down to what size will such an approach prevent overmatured M2 oocytes, without potentially reversing the gains in GV maturations achieved so far? That it is possible to retrieve mature MII oocytes from follicles as small as 10 mm has also been reported by Japanese colleagues, demonstrating smaller non-dominant follicles in modified natural cycles to be a good source of mature follicles.^22^ How small we can go, therefore, remains to be determined.

## Limitations of the study

This study has several limitations including, despite statistically significant findings, a relatively small study population. Even more importantly, this study was performed in a very adversely selected patient population for IVF which does not necessarily represent typical patient populations in most IVF centers. The findings reported here also do not necessarily apply to more favorably selected patients. Finally, as the number of good quality eggs and embryos usually also reflects clinical pregnancy and live birth chances,^23^ in judging the ability of oocytes to produce good quality embryos, this endpoint for the study can as of this point only be assumed to also represent pregnancy and live birth chances. Further, larger studies will be necessary to confirm this point.

## STAR★Methods

### Key resources table

**Table:** 

REAGENT or RESOURCE	SOURCE	IDENTIFIER
**Biological samples**		

10% SSS MHTF: HTF w/HEPES	LifeGlobal Group, Cooper Surgical Solutions, Målov, Denmark	GMHA-500
Serum Substitute Supplement (SSS)	Fuji Film Irvine Scientific, Santa Ana, CA	Cat#99197
Global Total	Cooper Surgical Solutions, Målov, Denmark	LGGT-030
Oocyte Maturation Media	Sage *In Vitro* Fertilization, CooperSurgical Solutions, Målov, Denmark	ART-1600-B

**Other**		

dehydroepiandrosterone (DHEA), 25 mg TID	Fertinatal, Ovaterra, New York, NY	N/A
300-450 I.U. of FSH product	manufacturer varies per patient insurance	N/A
150 I.U. of hMG product	manufacturer varies per patient insurance	N/A
Lupron, 4mg	Haas et al.^24^	N/A
human chorionic gonadotropin (hCG, 10,00 I.U.)	Haas et al.^24^	N/A

### Resource availability

#### Lead contact

Further information and requests for resources and reagents should be directed to and will be fulfilled by the Lead Contact, Norbert Gleicher (ngleicher@thechr.com or ngleicher@rockefeller.edu).

#### Materials availability

All materials and data are available from the lead contact upon reasonable request.

### Experimental model and study participant details

#### Institutional review board (IRB)

Every patient included in this study signed an informed consent that allowed the use of their medical record for research purposes, as long as the medical record remained confidential, and the identity of the patient was not revealed. As this study utilized our center’s anonymized electronic research databank, it fulfilled those criteria and was, therefore, approved by the Institutional Review Board of The Center for Human Reproduction by expedited review.

#### Patient population

The sex and gender of all participating patients were female and women. Ancestry, race, ethnicity, socioeconomic status, and individual ages are retrievable from the center’s anonymized electronic research database but were not analyzed for this study. As race and ethnicity are known to affect IVF outcomes,^25^ here-reported outcome data may differ between races and ethnicities, but such determinations will require a significantly larger study population.

It is important to emphasize that in this study, reported outcomes as of this moment cannot be generalized because they were obtained in very adversely selected infertility patients at ages where most IVF centers no longer treat infertile women with autologous oocytes. Moreover, investigated patient groupings were not large enough to allow for separate determinations by race and/or ethnicity. Therefore, here-reported observations may have different impacts on different races and/or ethnicities.

Retroactively investigating 150 consecutive IVF cycles performed at our center during 2021–2022, only donor-egg cycles and cycles in which no oocytes were retrieved were excluded. A total of 863 oocytes were retrieved in 103 women in 150 IVF cycles at median age 43.0 (range 32–52 years). Reflecting their poor prognosis beyond their advanced ages, median FSH and AMH were 9.0 mIU/mL and 0.643 ng/mL, respectively. Women under age 40 produced 265 oocytes, 161 M2s, 47 M1s, and 57 GV oocytes. Between ages 40–45 years, women produced 544 oocytes, 375 M2s, 89 M1s, and 80 GVs. Finally, above age 45, 54 oocytes were obtained, 35 M2s, 9 M1s, and 10 GVs.

### Methods details

#### IVF cycle stimulation protocol

Whatever the underlying cause, if androgens are low and/or if their SHBG is abnormally high, IVF cycles are initiated only after androgens and SHBG have normalized after pre-supplementation with dehydroepiandrosterone (DHEA, 25 mg TID, Fertinatal, Ovaterra, New York, NY) for at least 6–8 weeks prior to IVF cycle start.^26^

Ovarian stimulation in almost all cases involved 300–450 I.U. of an FSH product and 150 I.U. of an hMG product (manufacturer varies per patient insurance). With especially low ovarian reserve, patients also receive clomiphene citrate (100 mg) for 5 days, starting on day-2 of menses.

#### Highly individualized egg retrieval (HIER)

In contrast to most IVF centers which trigger IVF cycles at lead follicle sizes between 18 and 22 mm, our center since 2017 practices HIER.^4^^,^^5^ This means that, primarily based on age and other cycle parameters, patients are ovulation-triggered at much smaller lead follicle sizes. How lead follicle sizes at ovulation trigger changed with advancing age in the here-presented study, is demonstrated in Table 1, revealing a significant decline of average lead follicle sizes at ovulation trigger from 18.4 ± 3.4 mm below age 40 to 14.4 ± 3.3 mm above age 46 (p < 0.001).

Since triggers are given early, the risk of premature spontaneous ovulation almost no longer exists. Consequently, HIER cycles do not require either GnRH agonists or antagonists during hyperstimulation with gonadotropins to prevent premature ovulation. Here-investigated women, therefore, received neither.

#### Dual trigger

As initially reported in poor prognosis patients,^24^ and more recently reaffirmed in a larger prospectively randomized study,^27^ dual trigger to induce ovulation with human chorionic gonadotropin (hCG, 10,00 I.U.) and gonadotropin-releasing hormone antagonist (Lupron, 4 mg) appears to improve oocyte yields. Patients in this study, therefore, received dual triggers.

#### Embryology

Upon retrieval, oocyte-cumulus complexes (OCC) were rinsed in standard media [10% SSS MHTF: HTF w/HEPES, LifeGlobal Group, GMHA-500, Cooper Surgical Solutions, Målov, Denmark; Serum Substitute Supplement (SSS), Fuji Film Irvine Scientific, 99193, Santa Ana, CA] to remove follicular fluid and blood. The OCCs were trimmed and cultured for approximately 2 h prior to denudation. Once denuded, each oocyte stage of maturation was determined through the presence or absence of a germinal vesicle or polar body. M2 oocytes have one polar body between the ooplasm and the zona pellucida, M1 oocytes lack a polar body and germinal vesicle, and GV oocytes demonstrate germinal vesicles in the cytoplasm (Figure 1).

#### M2 oocyte processing

Following retrieval, M2s underwent intracytoplasmic sperm injection (ICSI) following denudation and were then placed in Global Total Media (Global Total, LifeGlobal Group, LGGT-030, Cooper Surgical Solutions, Målov, Denmark) for culture until the time of transfer on Day 3.

#### M1 and GV oocyte processing

As previously reported,^1^ immature M1 and GV oocytes underwent rescue *in vitro* maturation (IVM) in oocyte maturation media overnight (Oocyte Maturation Media, ART-1600-B, Sage *In Vitro* Fertilization, CooperSurgical Solutions, Målov, Denmark). They, however, were reexamined for maturation following ICSI of M2 oocytes: If by then matured, they immediately underwent ICSI. Those still immature were incubated overnight and reevaluated the following morning for maturation. Those that had matured immediately underwent ICSI and were placed into Global Total media (LifeGlobal Group, LGGT-030, CooperSurgical Solutions, Målov, Denmark) for culture until the time of transfer on Day 3.

#### Outcomes: Transferrable day-3 embryos

This study defined oocyte quality as the ability of a patient’s oocytes to produce transferrable day-3 embryos. On Day 3, embryo grading was completed by accessing the number of blastomeres, the evenness of the blastomeres, and the percentage of fragmentation. To be considered transferrable, an embryo on day-3 had to have at least 6–8 blastomeres if it underwent ICSI on retrieval day, and at least 2 blastomeres if ICSI was performed the following morning. Moreover, only embryos with grades A, B, or C for blastomere evenness and minimal fragmentation were considered transferable.

### Quantification and statistical analysis

Differences between age groups were compared with an ANOVA and, where appropriate, with the non-parametric Kruskal-Wallis test. A p-value of <0.05 was considered statistically significant. All statistics were performed by the center’s statistician (S.D.) with SAS version 9.4.

## Data Availability

Data All data reported in this paper will be shared by the lead contact by reasonable request. Code This paper does not report original code. All other items Any additional information required to reanalyze the data reported in this paper is available from the lead contact upon request.
